# The Validity and Value of Self-reported Physical Activity and Accelerometry in People With Schizophrenia: A Population-Scale Study of the UK Biobank

**DOI:** 10.1093/schbul/sbx149

**Published:** 2017-10-24

**Authors:** Joseph Firth, Brendon Stubbs, Davy Vancampfort, Felipe B Schuch, Simon Rosenbaum, Philip B Ward, Josh A Firth, Jerome Sarris, Alison R Yung

**Affiliations:** 1 NICM, School of Science and Health, University of Western Sydney, Sydney, Australia; 2 Division of Psychology and Mental Health, Faculty of Biology, Medicine and Health, University of Manchester, Manchester, UK; 3 Physiotherapy Department, South London and Maudsley NHS Foundation Trust, London, UK; 4 Health Service and Population Research Department, Institute of Psychiatry, Psychology and Neuroscience, King’s College London, London, UK; 5 Department of Rehabilitation Sciences, KU Leuven, Leuven, Belgium; 6 Department of Neurosciences, UPC KU Leuven, Leuven, Belgium; 7 Hospital de Clínicas de Porto Alegre, Porto Alegre, Brazil; 8 Centro Universitário La Salle, Canoas, Brazil; 9 Black Dog Institute, Randwick, Australia; 10 School of Psychiatry, University of New South Wales, Sydney, Australia; 11 Schizophrenia Research Unit, Ingham Institute of Applied Medical Research, Liverpool, Australia; 12 Department of Zoology, Edward Grey Institute, University of Oxford, Oxford, UK; 13 Department of Psychiatry, University of Melbourne, The Melbourne Clinic, Melbourne, Australia; 14 Greater Manchester Mental Health Foundation Trust, Manchester, UK

**Keywords:** exercise, psychosis, psychotic disorders, aerobic, cardiovascular, metabolic, accelerometer

## Abstract

**Background:**

Previous physical activity (PA) research in schizophrenia has relied largely upon self-report measures. However, the accuracy of this method is questionable. Obtaining accurate measurements, and determining what may influence PA levels in schizophrenia, is essential to understand physical inactivity in this population. This study examined differences in self-reported and objectively measured PA in people with schizophrenia and the general population using a large, population-based dataset from the UK Biobank.

**Methods:**

Baseline data from the UK Biobank (2007–2010) were analyzed; including 1078 people with schizophrenia (54.19 ± 8.39 years; 55% male) and 450549 without (56.44 ± 8.11; 46% male). We compared self-reported PA with objectively measured accelerometry data in schizophrenia and comparison samples. We also examined correlations between self-report and objective measures.

**Results:**

People with schizophrenia reported the same PA levels as those without, with no differences in low, moderate, or vigorous intensity activity. However, accelerometry data showed a large and statistically significant reduction of PA in schizophrenia; as people with schizophrenia, on average, engaged in less PA than 80% of the general population. Nonetheless, within the schizophrenia sample, total self-reported PA still held significant correlations with objective measures.

**Conclusions:**

People with schizophrenia are significantly less active than the general population. However, self-report measures in epidemiological studies fail to capture the reduced activity levels in schizophrenia. This also has implications for self-report measures of other lifestyle factors which may contribute toward the poor health outcomes observed in schizophrenia. Nonetheless, self-report measures may still be useful for identifying how active individuals with schizophrenia relative to other patients.

## Introduction

Lack of physical activity (PA) has been identified by the World Health Organization as one of the top-five causes of mortality worldwide.^1^ Physical inactivity is also strongly associated with the other main risk factors for premature mortality, including hypertension, hyperglycemia, and obesity,^1^ and increases the risk of cardio-metabolic disorders, such as diabetes and heart disease.^2^ Indeed, recent epidemiological evidence from European countries indicates that physical inactivity is responsible for twice as many deaths as obesity each year.^3^ Along with cardio-metabolic risk, physical inactivity is associated with lifetime incidence of common mental disorders, such as depression^4^ and anxiety.^5^ Furthermore, high levels of PA may contribute to a more favorable course of illness among those with schizophrenia,^6^ reducing both the psychiatric symptoms and cognitive dysfunction associated with this condition.^7,8^

Despite the clear benefits of exercise for both physical and mental health, a recent meta-analysis of 35 studies conducted across 10 different countries suggested that people with schizophrenia may be significantly less active than the general population,^9^ and experience multiple barriers to participate in regular exercise.^10^ Recent worldwide data have demonstrated that cardiovascular diseases are associated with a greatly increased premature mortality in those with schizophrenia^11^ compared to the cardiovascular diseases in the general population. This is also evident in the United Kingdom, where people with schizophrenia have some of the worst metabolic health and premature mortality recorded worldwide.^12,13^ These health inequalities continue to grow in this patient group.^14^ Although this may be due to lifestyle factors, no studies have compared PA levels of people with schizophrenia in the United Kingdom to the general population.^9^

Furthermore, the majority of previous studies of PA in schizophrenia have relied upon self-report measures,^9^ the validity and value of which remains largely unknown for this population. Previous work has suggested that these measures may have low reliability and show substantial differences to ”gold standard” objective measures (ie, accelerometry) in people with schizophrenia.^15^ Thus, further research is required to obtain accurate estimates of PA in people with schizophrenia, establish the usefulness of self-report measures, and determine which factors may influence PA estimates. Indeed, even internationally, there is only a small number of population-scale studies which have examined PA in people with psychotic disorders, and all of these have relied entirely on self-report data.^16,17^

Therefore, the aim of this study was to examine differences in both self-reported and objectively measured levels of PA in people with schizophrenia and the general population using a large, population-based dataset from the UK Biobank. We also aimed to examine the epidemiological and clinical utility of self-report measures in people with schizophrenia through assessing the extent to which self-reported PA correlates with objective measurements.

## Methods

We conducted a cross-sectional analysis of data from the baseline assessments for the UK Biobank study, collected between 2007 and 2010. The UK Biobank is a population-scale, epidemiological study assessing how various health-related outcomes relate to lifestyle, environmental, and genetic factors.^18^ Invitations were mailed to around 9.2 million residential addresses in the United Kingdom, which successfully recruited 502664 adults aged between 37 and 73. Prospective participants attended their closest dedicated assessment center of the 22 located throughout the United Kingdom. Here, they provided informed consent and undertook an extensive battery of computerized questionnaires, physical health assessments, and in-person interviews with research staff. The full protocol and data collection processes for the UK Biobank are available elsewhere.^19^

Specific datasets from the UK Biobank are released to applicants for the purpose of investigating prespecified research questions, following review and approval from the UK Biobank’s Access Sub-Committee. This particular study was approved by the committee on October 4, 2016, and is covered under the generic ethical approval from the NHS Research Ethics Committee (Ref. 11/NW/0382).

### Participants

The UK Biobank is linked to NHS hospital admission data, enabling access to recorded clinical diagnoses. For the purposes of this study, we classified the schizophrenia sample as participants in the UK Biobank with a recorded primary or secondary ICD-10 diagnosis of any nonaffective psychotic disorder, including schizophrenia and schizotypal disorders (disease classes F20–F29). The comparison sample was all of the UK Biobank participants with no recorded history of schizophrenia (or any other nonaffective psychotic disorder).

### PA and Additional Assessments

PA was assessed over a typical week using 2 methods. First, all participants provided a self-report measure of PA by completing a touchscreen version of the IPAQ short-form, which has been used extensively in both healthy populations and patients with schizophrenia.^9,20^ This questionnaire assesses PA over the previous week by asking participants about the length and frequency of their engagement in (1) walking, (2) moderate-intensity activity, and (3) vigorous-intensity activity. Scores for each of these different modalities of exercise were then used to calculate a single score for “total PA” over the previous week, in line with IPAQ guidelines, expressed as “metabolic equivalent minutes” (MET minutes) of activity per week.^21^ As these estimates rely upon people’s recall of activity over the previous week, participants with chronic neurological diseases which impair memory were excluded from all analyses (supplementary table 1). Age, gender, and other sociodemographic characteristics were also collected from the computerized questionnaire completed at the UK Biobank assessment centers. Body mass index (BMI) was calculated from height and weight measurements taken by a research assistant on-site during the physical health assessments.

PA in a subsample of participants at a later date was further measured using accelerometry; the most frequently used objective assessment of PA for epidemiological studies.^22,23^ A total of 236519 participants of the UK Biobank (ie, all those who had provided a current email address) were invited to take part in a 1-week accelerometer study. Of these, 106053 signed-up to wear a PA monitor (44.8%) for 1 week. Those who signed-up via email were mailed an “Axivity AX3 wrist-worn triaxial accelerometer” by post, programmed to capture triaxial acceleration data over 5 s epochs for the period of 1 week, at 100 Hz and a dynamic range of ±8 *g*. Nonwear time was defined as stationary periods (ie, where all 3 axes had a standard deviation of less than <13.0 mg) lasting for 60 min or longer.^24^ Identical instructions were provided to each participant; informing them to secure the device around their dominant wrist, wear it continuously for 7 days while engaging in their regular day-to-day activities, and then return the device to the UK Biobank assessment centre via post, using the provided prepaid envelope. The accelerometry scores used in this study were calculated from the raw accelerometer data using a rigorous data processing procedure, published elsewhere.^24^ This was computed as “mean vector magnitude,” which takes into account both the time and intensity of motion, in order to provide a summary score representing an average PA level over a given time period (in this case, one week). Participants who returned poorly calibrated devices (<0.1% of participants) were unable to be included in the analyses. Overall compliance was high, as 80.6% of participants wore the device for >150/168 h. However, 6978 participants (6.7% of sample) who had insufficient device wear time (<72 h) to accurately estimate average PA were excluded from analyses.^24^

### Statistical Analyses

All analyses were conducted in the statistical software R (version 3.1.2). To reduce the impact of anomalous data (ie, and those deemed due to error), we removed those which reported extremely high levels of activity and appeared as outliers (ie, the top 0.5%—supplementary figure S1) and those with self-report data and accelerometer data which were equal to zero weekly activity whatsoever (>0.1%). Self-reported activity data were log transformed to resolve the left skew of the distribution (supplementary figure S1). Between-subjects *t*-tests and Wilcoxon tests were then used to summarize the raw mean differences in self-reported PA (for low intensity, moderate intensity, vigorous intensity, and total METs) and objectively measured activity (accelerometer mean vector magnitude) between schizophrenia and comparison samples. Following this, linear mixed models controlling for age, gender, and BMI as fixed effects, along with ethnicity and Biobank testing location as random effects, and setting the response variables as the activity measure of interest, were performed to examine between-group differences after controlling for these potentially confounding variables.

We then aimed to quantify the magnitude of deficits in PA among people with schizophrenia. Firstly, for both self-report and accelerometry-calculated activity separately, we calculated which quantile each individual with schizophrenia fell into within the total distribution of the general population. By comparing the average (and 95% range) of these quantile scores for self-reported activity to that of accelerometry activity, this provides an intuitive description of the extent to which people with schizophrenia differ from the general population depending on the activity measure used.

Finally, we aimed to establish how well self-reported activity within people with schizophrenia aligned their accelerometry activity. We assessed the correlation between self-reported and accelerometry-measured PA levels within the schizophrenia sample using both ranked scores within the sample (Spearman’s correlation) as well as their percentile score within the general population (Pearson’s correlation). This allowed us to assess the utility of self-report measures as a tool for comparing individuals with schizophrenia on the basis of their PA levels.

## Results

A total of 451627 UK Biobank subjects matched eligibility criteria and had provided at least one aspect of PA information to be included in at least part of this study. The patient sample consisted of 1078 participants with nonaffective psychotic disorders, diagnosed according to ICD-10 criteria. The mean age at study enrolment assessment was 54.19 years (SD = 8.39, range = 40–70) and were 55% male. The total comparison sample consisted of 450549 participants with no recorded history of schizophrenia (or any other nonaffective psychotic disorders). In the comparison group, the mean age was 56.44 years (SD = 8.11, range = 38–73) and were 46% male. The sociodemographic characteristics of patient and comparison samples for different levels of the analysis are displayed in table 1.

**Table 1.** Demographics and Physical Activity Levels in Schizophrenia and General Population

Schizophrenia Sample				Comparison Group		
	Total, *n*	Raw Mean	SD	Total, *n*	Raw Mean	SD
Age	1078	54.19	8.39	450549	56.44	8.11
Gender	1078	55% male		450549	46% male	
BMI	1059	28.61	5.79	448604	27.3	4.69
Low intensity PA	1018	359.04	535.23	414558	413.72	581.75
Moderate PA	733	294.84	588.54	359224	321.47	479.22
Vigorous PA	472	164.38	508.84	263435	149.49	237.72
Total METs	406	3724.47	3802.2	227711	3843.4	3860.5
Accelerometry score^a^	84	23.30	9.47	101516	27.42	9.47

*Note:* BMI, body mass index; METs, metabolic equivalent units; PA, physical activity (minutes per week); SD, standard deviation.

^a^Vector magnitude.

### PA in Schizophrenia and Comparison Samples

**Fig. 1.**
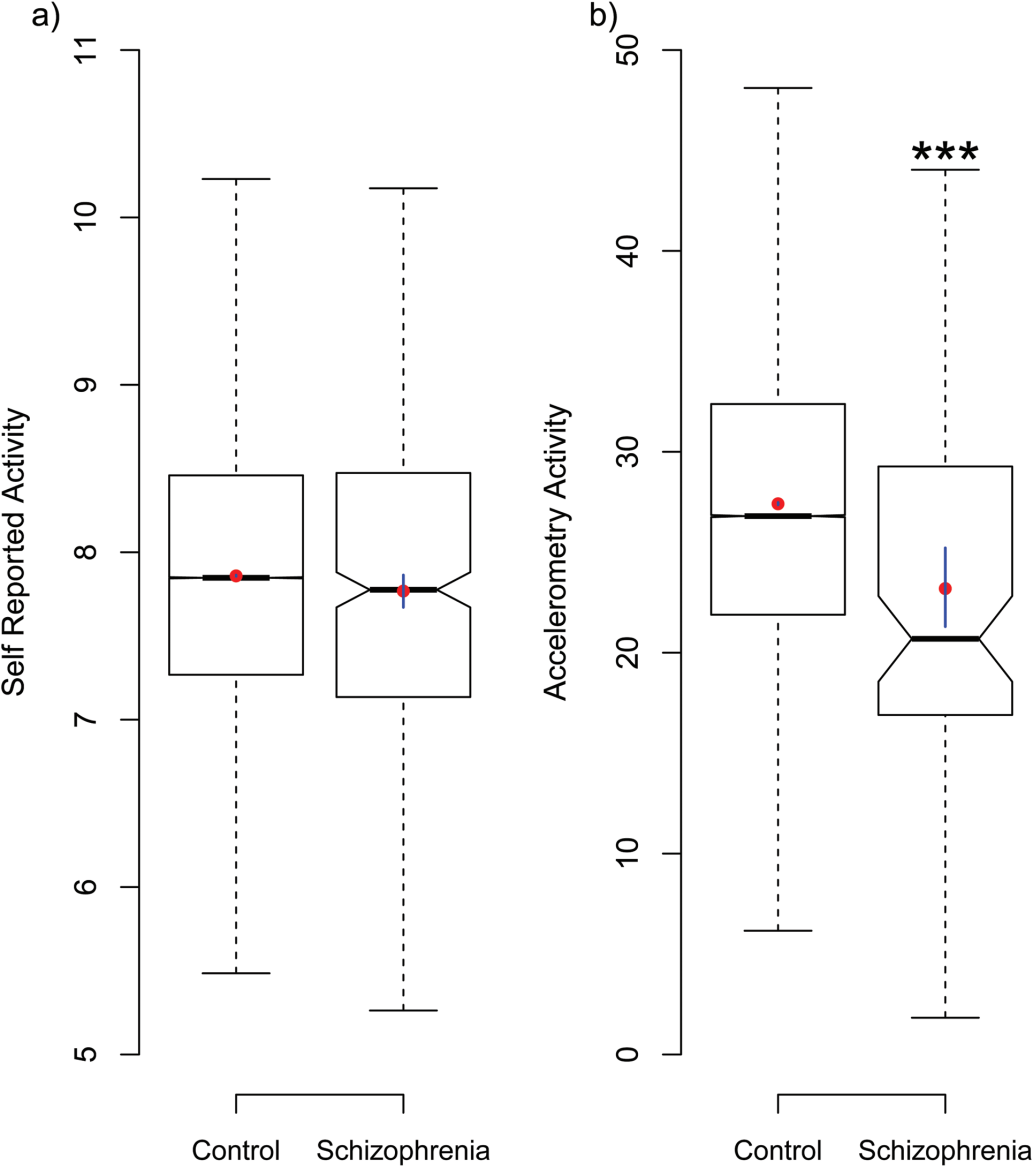
Summary of (a) self-reported activity (log transformed METs) levels and (b) accelerometry-measured activity, for those with a recorded diagnosis of schizophrenia (“Condition”) and those without (“Control”). Within the boxplots, boxes show the interquartile range (IQR), whiskers indicating the range (excluding values 1.5 times outside of IQR), mid-lines denote the median and notches show the estimated 95% confidence around the median. The red circular points show the sample mean and the vertically transecting blue lines show the 95% confidence interval (estimated using 10000 bootstrap samples). Asterisk indicates a significant difference as *P* < .001 (see “Results” section).

Levels of self-reported and objectively measured PA in schizophrenia and comparison samples are displayed in table 1. Simple statistical tests comparing levels of total self-report PA (shown in figure 1a) found no differences between people with schizophrenia (mean = 3724 ± 3802 METs per week) and those without (mean = 3843 ± 3861 METs per week) (*t*-test: *t* = 1.81, df = 406.16, *P* = .07, Wilcox *P* = .15). This was also confirmed by linear mixed models controlling for age, gender, BMI (as fixed effects) and ethnicity and testing location (as random effects) (LMM coeff = −0.45, SE = 0.45, *t* = 1.01, *P* = .312). We also performed separate analyses to compare schizophrenia and comparison samples for levels of low, moderate, and vigorous intensity activity. These analyses found no differences in any type of self-reported activity levels for those with schizophrenia compared to those without (table 1 and supplementary figure S3).

However, objectively measured PA (ie, accelerometry data) showed that people with schizophrenia engaged in significantly less weekly activity (mean vector magnitude = 23.3 ± 9.74) than those without (mean vector magnitude = 27.42 ± 9.47). As shown in figure 1b, this difference was statistically significant when comparing the 2 groups directly (*t*-test: *t* = 4.09, df = 83.14, *P* < .0001, Wilcox *P* < .0001) as well as when controlling for potentially influential variables (LMM coeff = −4.585, SE = 0.981, *t* = −4.67, *P* < .001). Additionally, we carried out sensitively analyses considering only those participants who had provided both sufficient data for both self-reported total activity scores and accelerometry totals (to ensure the above pattern was not related to sample size) and found the same results (supplementary figure S2). Finally, we also performed sensitivity analyses using a patient sample including only those with a diagnosis of schizophrenia, schizotypical disorder or schizoaffective disorder (ICD-10 codes F20, F21, and F25: 78% of total sample). This found the same result as the primary analyses; with accelerometry scores showing large, significant differences between schizophrenia and comparison samples (LMM coeff = −0.104, SE = 0.057, *t* = −1.826, *P* < .068), but with self-report measures failing to find a significant difference in total PA between groups (LMM coeff = −4.762, SE = 1.312, *t* = −3.630, *P* < .001).

This divergence between in self-reported and accelerometry activity levels was clearly demonstrated when assessing which quantile the schizophrenia sample’s score fell into with the general population: When using self-report PA totals, the median average scores from the schizophrenia sample fell into the 47th percentile for self-reported activity, with a 95% confidence interval ranging from 40th percentile to the 52nd percentile. In contrast, when using accelerometry averages, people with schizophrenia fell into the bottom 20% of the general population, with a 95% confidence ranging from 15% to 35%. Thus, while self-reported measures generally place people with schizophrenia as functionally equivalent to those without, the accelerometry scores show that, on average, people with schizophrenia engage in less PA than 80% of the general population.

**Fig. 2.**
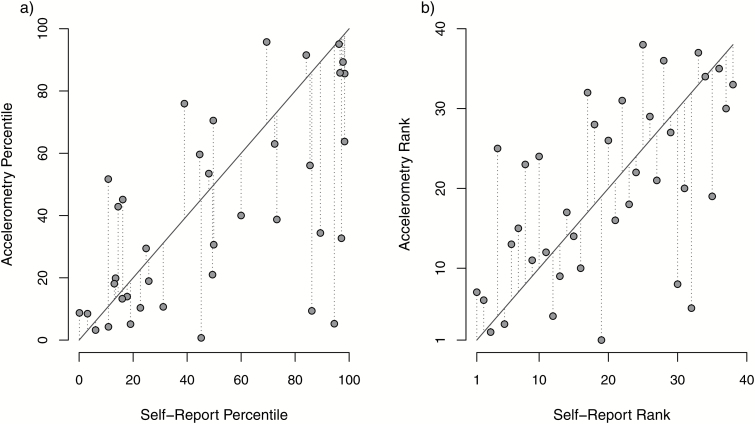
The correlation between self-reported activity scores and accelerometry activity scores when measured as either (a) the percentile within the general population or (b) the rank within schizophrenia. Points show the raw data scores, solid line shows 1:1 fit and dotted lines indicate the distance between the raw data points and the 1:1 fit.

Although self-report measures failed to provide an accurate indication of levels of PA in schizophrenia compared to the general population, our correlational analyses within the schizophrenia sample found that self-report measures are useful for comparing levels of PA among individuals with schizophrenia: First, when looking only within the schizophrenia sample, individual’s rank on self-report accelerometry measures showed a moderate correlation with their ranked accelerometry activity score (Spearman’s correlation: 0.57, S = 3946, *P* < .001, figure 2b). Similarly, when looking at where each individual with schizophrenia fell with regards to the general population, the percentile they fell into from their self-reported scores was highly correlated with their accelerometry activity percentile score (Pearson’s correlation: 0.59, *t* = 4.37, *P* < .001, figure 2a). We also sought to examine if any inter-individual factors among people with schizophrenia predicted their accuracy of self-report vs. objectively measured activity means. After calculating an activity divergence score within people with schizophrenia (simply their self-reported percentile minus their accelerometry percentile), we did not find any evidence that age, gender, or BMI of people with schizophrenia predicted the difference between their self-reported activity and accelerometry measure (*t* < 0.2, *P* > .05). Therefore, the relationship between relative self-reported and accelerometry scores is expected to be reasonably consistent across different classes of individuals.

## Discussion

This, to our knowledge, is the first study to examine differences in self-report and objectively measured PA between people with schizophrenia and those without in a population-scale dataset (ie, the UK Biobank). We also present the first large-scale research on PA levels among people with schizophrenia in the United Kingdom, who have some of the worst premature mortality and metabolic health outcomes measured across the world,^12,13^ which is largely attributed to lifestyle factors (such as PA). Across 1078 people with a lifetime diagnosis of schizophrenia (and schizophrenia-like disorders), and 450549 people without, we show that schizophrenia is not associated with any differences in self-report PA (as measured with an adapted version of the IPAQ). The data clearly indicated that people with schizophrenia matched the same self-reported scores of the average population for all classes of PA, including low-intensity, moderate, and vigorous exercise, along with total weekly levels. Therefore, this self-report data alone would indicate that the health inequalities displayed by this population could not be ascribed to reduced levels of PA, and suggests that to experience further benefits from PA (for physical and mental health), people with schizophrenia would actually have to engage in more than the average level of exercise.

However, our analysis of accelerometry data (the “gold-standard” objective measures of PA for epidemiological studies^22,23^) showed that in fact, people with schizophrenia do show reduced levels of tracked activity compared to those without. This was a large and robust difference, with 95% of people with schizophrenia falling within the bottom 15%–35% of the general population. This large difference was equally apparent when using models to additionally control for age, gender, BMI, and sociodemographic variation. Therefore, this study shows that while self-reported data in population studies would suggest no PA deficit for people with schizophrenia, accelerometry data actually strongly supports the relationship between schizophrenia and insufficient PA.^9^

Given that PA is one of the strongest risk factors for premature mortality worldwide,^1^ and people with schizophrenia die around 15–25 years younger than the general population (largely due to cardiometabolic health conditions),^12^ exercise interventions present an immediate and obvious opportunity for improving the physical health outcomes of this population.^14,25^ However, the means and opportunity to improve the physical health of this group through increasing PA would be unknown if self-report data were relied upon.

Furthermore, in the general population, physical inactivity has also been shown to be associated with poor mental health (ie, depression and anxiety) and impairments in cognitive functioning^4,26^; both of which are prevalent and enduring facets of schizophrenia. Thus, increasing PA may also improve mental health outcomes, and reduce the personal and societal burden of the disorder.^27^ Indeed, randomized controlled trials have shown that exercise interventions can significantly reduce symptoms and cognitive dysfunction in people with schizophrenia.^7,8^ However, efforts are still required to implement PA as a core aspect of clinical treatment for this disorder, and to evaluate the impact on physical and mental health from a service-level perspective.^27,28^

Previous studies have established that self-report tools such as the IPAQ can be useful for identifying the most/least active patients within a schizophrenia sample.^15^ Our findings support this, but also show that these self-report measures are unsuitable for studying differences in PA between people with/without schizophrenia in population-level studies, significantly over-estimating PA levels in the target population, and failing to capture large, significant differences which exist between samples. The inaccuracy of self-report PA in people with schizophrenia may also extend to measurement of other relevant constructs, such as diet, medication adherence and social interactions. Along with developing more accurate self-report measures for this population,^29^ epidemiological studies should consider implementing new tracking methods to measure PA. One promising option is mobile phones, which provide an ever-increasing range of “mHealth” options for actively and passively measuring PA, along with other lifestyle variables, relevant to psychiatric populations.^30–32^ However, it must be considered that the clinical usefulness of these new technologies is ultimately reliant upon patient compliance.^33^ Although various strategies have proven effective for promoting compliance to traditional accelerometer devices in people with schizophrenia,^34^ optimal techniques for engaging patient in mobile health monitoring and intervention have yet to be determined. Nonetheless, given the broad acceptability and adoption of these technologies among people with schizophrenia,^35,36^ mobile devices could ultimately provide a new platform for identifying and intervening in low PA (and other adverse lifestyle behaviors) for this patient group.

One limitation of this study is the relatively limited size of the schizophrenia sample in comparison to the general population; as the 1078 patients identified fell short of the estimated 1% which would be expected from the total sample. This is because the UK Biobank study did not systematically assess/interview participants for schizophrenia or related disorders. Instead, the information available for identifying patients with schizophrenia was through UK Biobank links to diagnostic records. Nonetheless, this methodology also adds strength to the results, by assuring that the target sample did indeed have a ICD-10 diagnosis of schizophrenia (or related disorders).

The lack of psychiatric evaluation data also meant we were unable to examine how the clinical characteristics of individuals with schizophrenia influence the accuracy of self-report measures. In this study, we investigated the sociodemographic factors (eg, age, gender) that caused divergence between self-report and objective measures for the schizophrenia sample; finding that none of these particular factors influenced this, thus indicating self-report measures are equally inaccurate across different classes of patients. However, due to a lack of clinical data in the UK Biobank, we were unable to explore the impact of other putative factors, such as symptom severity, illness duration, and cognitive dysfunction. Future research should aim to determine which factors influence the accuracy of self-report measures in this population (for PA and for other constructs), to inform the clinical application of these tools (by identifying which individuals self-report measures are/are not suitable for), along with providing new insights into what causes the observed inaccuracies in self-report measures.

A further limitation is that the accelerometry data were collected at a different time-point to self-reported PA, and thus some differences in weekly averages would be expected. Nonetheless, our findings that self-reported activity held significant and moderately strong correlations with accelerometry scores indicates weekly PA totals were reasonably consistent over the data collection period. Finally, in this study, accelerometry data was only available for “total weekly PA,” as we used accelerometry scores derived from previous data processing studies of the UK Biobank (rather than raw data).^24^ Given that the self-report data showed equivalence between people with schizophrenia and general population across all classes of low intensity, moderate and vigorous activity, it would be interesting to objectively assess where the differences in activity for people with schizophrenia actually arose from. Furthermore, the role of sedentary behavior should not be neglected. This can be considered an independent construct to PA/exercise in terms of individual’s engagement levels, and its impact on health.^37,38^ Previous meta-analyses have shown that people with schizophrenia engage in high levels of daily sedentary behavior, which is under-estimated by self-report measures.^39^

Future studies should aim to improve the accuracy of self-report measures, or increase the ubiquity of objective measures, to determine which types of PA are most reduced, and which types of sedentary behaviors are most increased, in people with schizophrenia. Future research should examine how deficits in specific intensities of activity relate to both cardiovascular health and psychiatric symptoms among people with schizophrenia. It should also be noted that this research was conducted in older age individuals, for both the schizophrenia and comparison samples. Since PA decreases per decade in both the general population^24^ and in people with psychosis,^40^ our findings may not generalize to younger people. Indeed, younger patients, in earlier stages of psychotic disorders, are generally more active, fitter and more likely to engage in moderate to vigorous exercise than older patients with long-term schizophrenia.^9,40–42^ Further research should aim to identify when differences in PA between people with schizophrenia and the general population first arise, to inform the development of targeted PA programs for improving the typically poor physical and mental health outcomes observed in this population.

## Supplementary Material

Supplementary data are available at *Schizophrenia Bulletin* online.

## Funding

S.R. is funded by a UNSW Scientia & NHMRC Early Career Fellowship (APP1123336). J.S. is funded by an NHMRC Research Fellowship (APP1125000). J.F. is funded by a Blackmores Institute Fellowship and an MRC Doctoral Training grant (P117413F07). B.S. is supported by the National Institute for Health Research (NIHR) Biomedical Research Centre at South London and Maudsley NHS Foundation Trust and King’s College London.

## Supplementary Material

Supplementary Information

Supplementary Figure S1

Supplementary Figure S2

Supplementary Figure S3

Supplementary Table 1
