# Supplementary material for: The Validity and Value of Self-reported Physical Activity and Accelerometry in People With Schizophrenia: A Population-Scale Study of the UK Biobank
Source: Schizophr Bull. 2017 Oct 24;44(6):1293–300. doi: 10.1093/schbul/sbx149 (PMC6192495; doi:10.1093/schbul/sbx149)
Supplement: Supplementary Information [file sbx149_suppl_supplementary_information.docx]

**Supplementary Information**

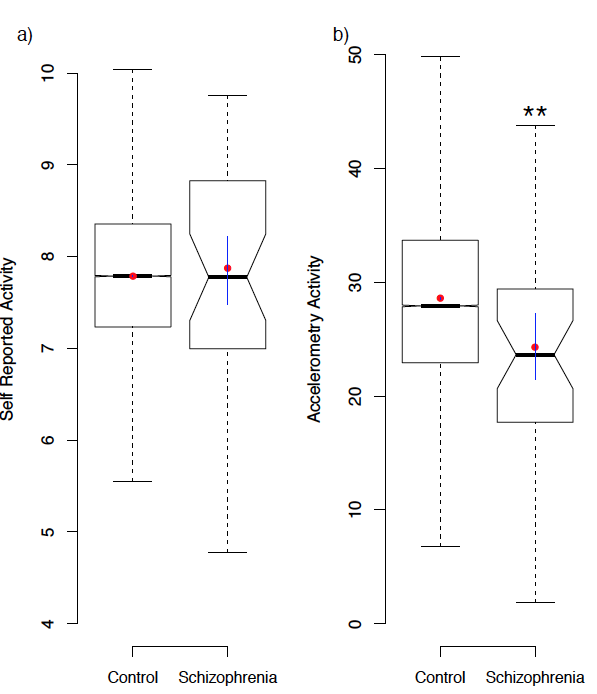


Figure S2. Following Figure 1 of main text but only including individuals that had full self-reported scores and accelerometry data (n=53738). Summary of (a) Self-reported activity (log transformed METs) levels and (b) Accelerometry-measured activity, for those with a recorded diagnosis of schizophrenia (‘Condition’) and those without (‘Control’). Within the boxplots, boxes show the interquartile range (IQR), whiskers indicating the range (excluding values 1.5times outside of IQR), mid-lines denote the median and notches show the estimated 95% confidence around the median. The red circular points shows the sample mean and the vertically transecting blue lines show the 95% confidence interval (calculated using 10000 bootstrap samples). No differences were found between the two groups for self-reported activity (t test: t=-0.42 , df=37 , p=0.68 , Wilcox p =0.86 ) including when controlling for potentially influential factors (see Methods) in linear mixed models (LMM coeff = 0.106, SE = 0.139, t = 0.761, p = 0.447). However, those suffering from schizophrenia showed significantly less accelerometry activity than non-schizophrenia (t test: t= 2.91 , df=37 , p=0.006 , Wilcox p = 0.003) and this difference was robust to controlling for other factors (LMM coeff =-5.440 , SE =1.51 , t = -3.611 , p<0.001).


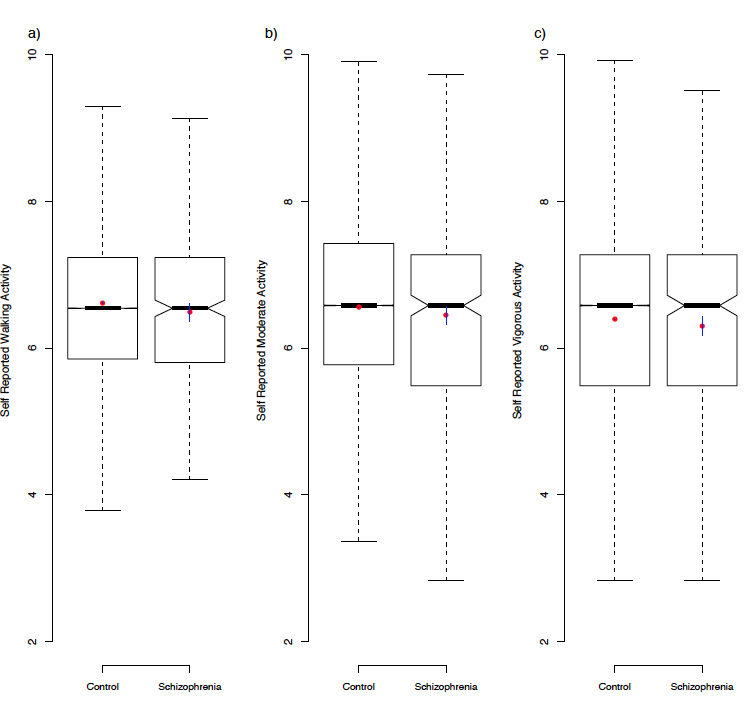


Figure S3. Following Figure 1 of main text but considering the three measures of self-reported activity levels. Summary of (a) Self-reported activity (log transformed METs) levels and (b) Accelerometry-measured activity, for those with a recorded diagnosis of schizophrenia (‘Condition’) and those without (‘Control’). Within the boxplots, boxes show the interquartile range (IQR), whiskers indicating the range (excluding values 1.5times outside of IQR), mid-lines denote the median and notches show the estimated 95% confidence around the median. The red circular points shows the sample mean and the vertically transecting blue lines show the 95% confidence interval (calculated using 10000 bootstrap samples). No differences were found between the two groups for any separate measure of self-reported activity (t test: t<2 , p>0.05 , Wilcox p>0.05 ) including when controlling for potentially influential factors (see Methods) in linear mixed models.
