# Supplementary figures and images for: The Validity and Value of Self-reported Physical Activity and Accelerometry in People With Schizophrenia: A Population-Scale Study of the UK Biobank
Source: Schizophr Bull. 2017 Oct 24;44(6):1293–300. doi: 10.1093/schbul/sbx149 (PMC6192495; doi:10.1093/schbul/sbx149)

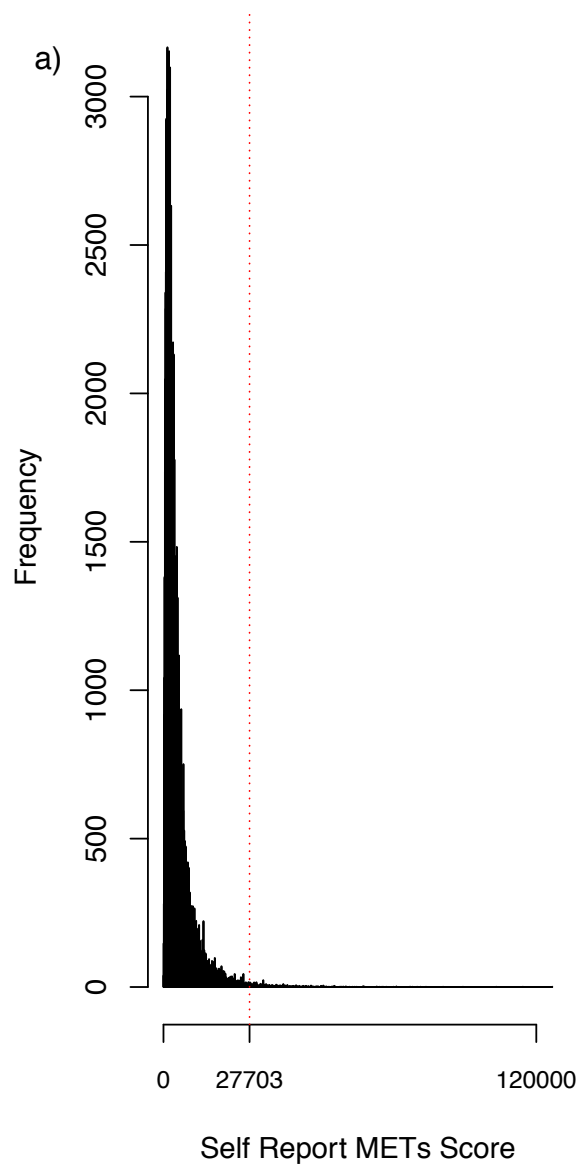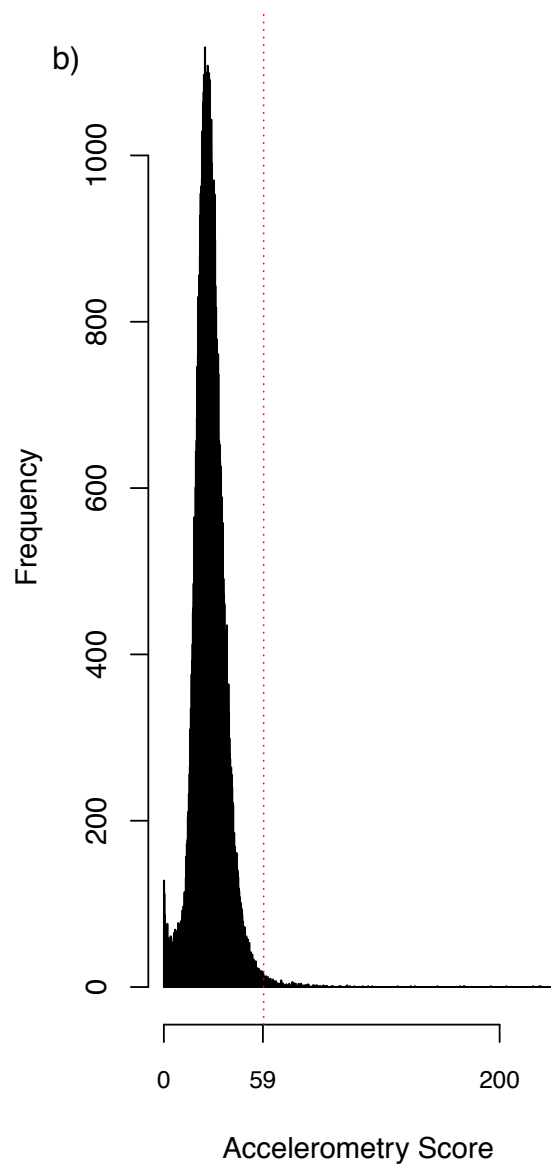

Supplement: Supplementary Figure S1 [file sbx149_suppl_supplementary_figure_s1.pdf]

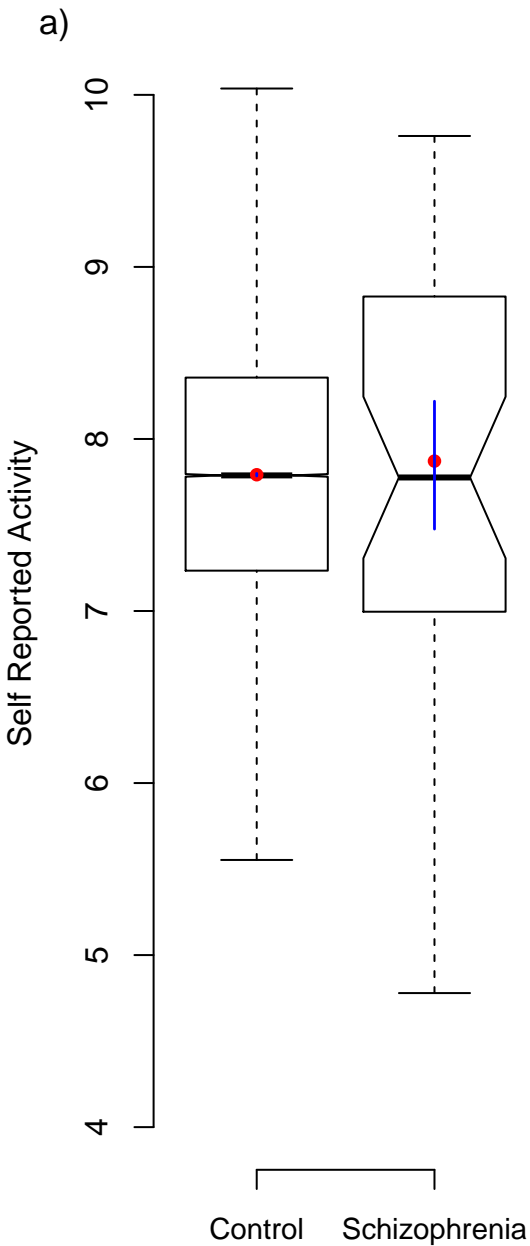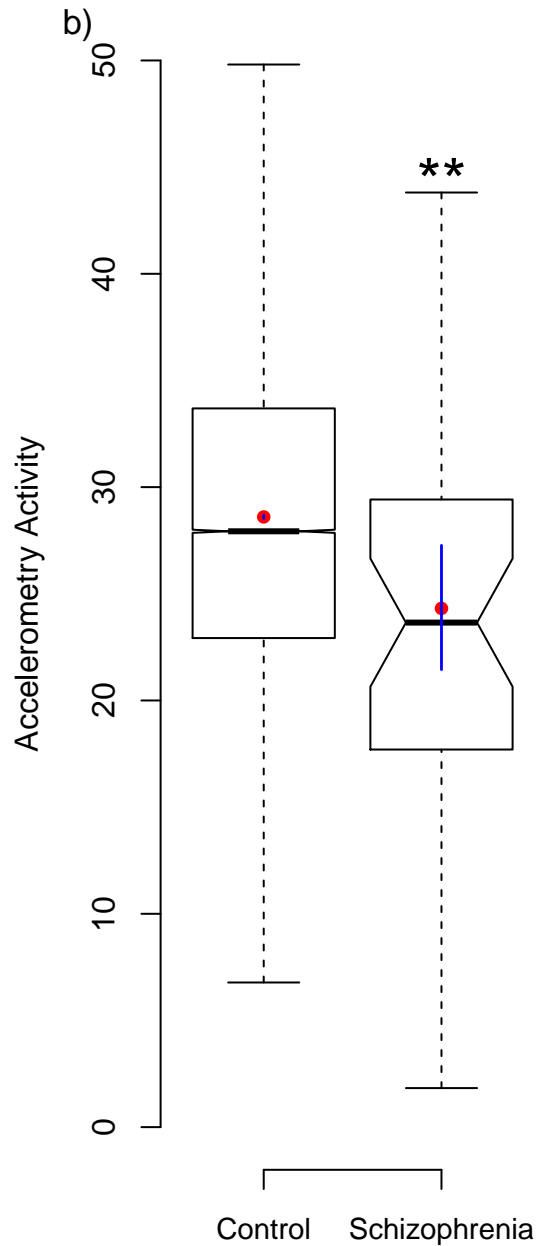

Supplement: Supplementary Figure S2 [file sbx149_suppl_supplementary_figure_s2.pdf]

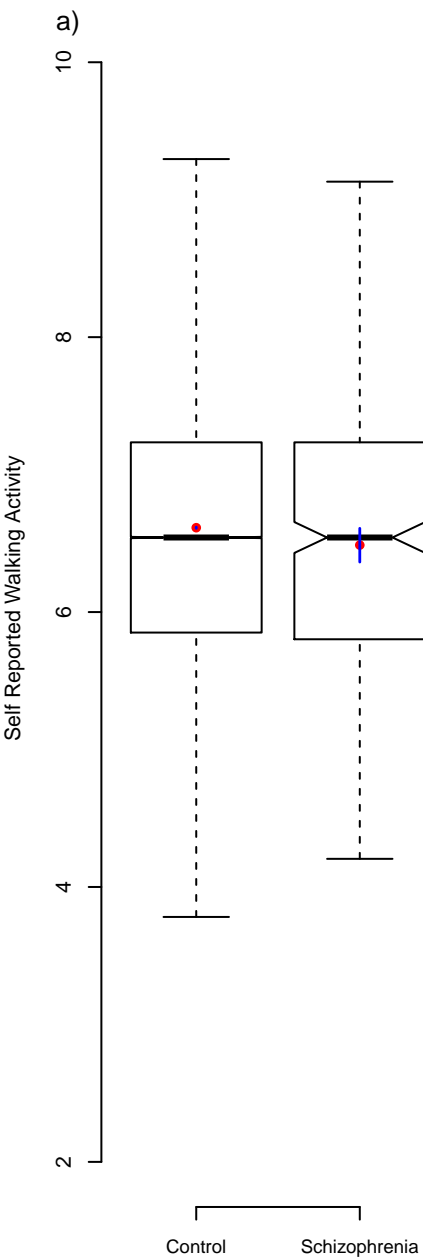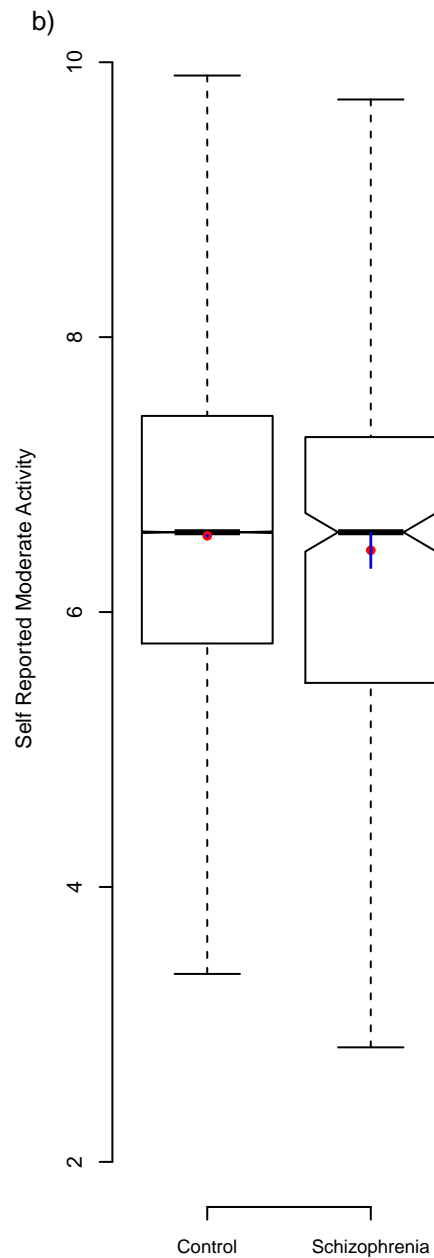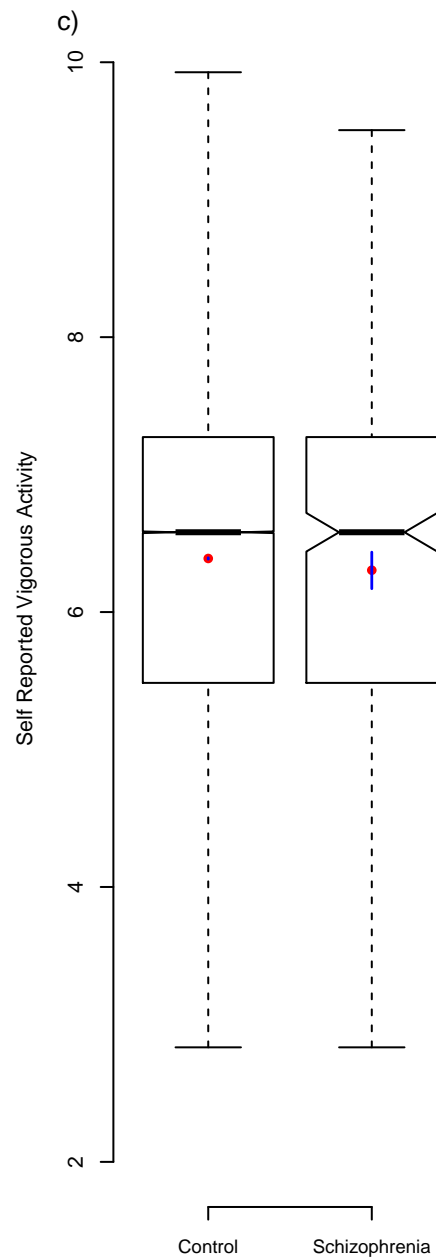

Supplement: Supplementary Figure S3 [file sbx149_suppl_supplementary_figure_s3.pdf]
