# Supplementary material for: The Validity and Value of Self-reported Physical Activity and Accelerometry in People With Schizophrenia: A Population-Scale Study of the UK Biobank
Source: Schizophr Bull. 2017 Oct 24;44(6):1293–300. doi: 10.1093/schbul/sbx149 (PMC6192495; doi:10.1093/schbul/sbx149)
Supplement: Supplementary Table 1 [file sbx149_suppl_supplementary_table_1.docx]

Supplementary Table 1. Neurological conditions which excluded participants from the study.

| UK Biobank Field Codes | **Field 6150** = 3  **Field 20001** = 1031 OR 1032  **Field 20002** = 1082 1083 1086 1524 1262 1397 1683 1245 1246 1491 1425 1433 1258 1263 1264 1266 1244 1583 1659 1259 1240 1434 |
| --- | --- |
| Condition titles | Brain cancer/primary malignant tumour  Brain haemorrhage  Brain/intracranial abscess  Cerebral aneurysm  Cerebral palsy  Chronic/degenerative neurological problem  Dementia/Alzheimer's disease/cognitive impairment  Encephalitis  Epilepsy  Head injury  Infection of nervous system  Ischaemic stroke  Meningeal cancer/malignant meningioma  Meningioma (benign)  Meningitis  Motor neurone disease  Multiple sclerosis  Neurological injury/trauma  Neuroma (benign)  Other demyelinating condition  Other neurological problem  Parkinson's disease  Spina bifida  Stroke  Subarachnoid haemorrhage  Subdural haematoma  Transient ischaemic attack |
